# SOP: emergency workup in patients with suspected acute bacterial meningitis

**DOI:** 10.1186/s42466-020-00098-6

**Published:** 2021-01-07

**Authors:** Susanne Dyckhoff-Shen, Uwe Koedel, Hans-Walter Pfister, Matthias Klein

**Affiliations:** 1 Department of Neurology, LMU Klinikum, Ludwig Maximilians University Munich, Marchioninistr 15, 81377 Munich, Germany; 2 Emergency Department, LMU Klinikum, Ludwig Maximilians University Munich, Marchioninistr 15, 81377 Munich, Germany

**Keywords:** Meningitis, SOP, Bacteria, CSF, Antibiotics

## Abstract

**Introduction:**

Despite antibiotic therapy, adjunctive treatment with dexamethasone, and care on modern intensive care units, bacterial meningitis remains a life-threatening disease with a high mortality and morbidity. One of most critical factors that influences outcome is a targeted quick but profound workup and early initiation of therapy in the Emergency Department. This standardized operating procedure was designed to guide physicians through the workup of patients with suspected acute bacterial meningitis.

**First steps:**

In patients with suspected community-acquired bacterial meningitis, the first steps aim at establishing a diagnosis and at starting empiric therapy without delay. Therefore, physicians need to seek for an early lumbar puncture that can be done safely without prior imaging if clinical signs that point at contraindications of a lumbar puncture are absent. Immediately after lumbar puncture, empiric therapy with ceftriaxone, ampicillin and dexamethasone should be started. In regions with a critical resistance rate of pneumococci against third generation cephalosporines, vancomycin or rifampicin need to be added.

**Comments:**

Clinical signs that are associated with intracranial conditions that are a contraindication for a lumbar puncture are severely decreased consciousness, new onset focal neurological signs, and epileptic seizures. If any of these clinical signs are present, cerebral imaging is recommended before lumbar puncture. Whenever lumbar puncture is delayed, empiric therapy needs to be begun before cerebrospinal fluid is obtained.

**Conclusion:**

Suspected acute bacterial meningitis is an emergency and requires attention with high priority in the emergency department to ensure a quick workup and early start of therapy.

## Introduction

Acute bacterial meningitis is a neurological emergency with a high mortality rate. Furthermore, a large proportion of survivors suffers from long term sequelae [1]. Worldwide, meningitis is one of the four most important contributors of disability due to neurological disorders [2]. The outcome in bacterial meningitis largely depends on a timely diagnosis, a prompt start of a targeted antibiotic therapy, and an early detection of complications [3, 4]. Thus, part of the battle’s outcome is decided within the first hour(s) after arrival of the patient at the hospital – in the emergency department. This standard operating procedure (SOP) is meant to guide physicians through targeted diagnostic emergency workup and focused empiric therapy in patients with suspected bacterial meningitis. The recommendations are based on the European Society of Clinical Microbiology and Infectious Diseases (ESCMID) guidelines and the guidelines of the German Neurological Society (DGN) on acute bacterial meningitis [3, 4]. Of note, the DGN guidelines are currently undergoing a revision process (expected date of publication: end of 2021) and single aspects of this SOP might then require reconsideration depending on the recommendations of the revised guideline.

## Definitions

### Meningitis

Meningitis is an infection of the meninges, the surrounding membranes of the brain and spinal chord. It is mainly caused by viruses or bacteria, the disease being more severe in the latter case.

### Meningoencephalitis

In meningoencephalitis, the infection is not only located along the meninges but has reached the brain itself.

## First steps

The critical first step in the emergency department is to recognize patients who show clinical signs that are consistent with acute bacterial meningitis. If patients with suspected acute bacterial meningitis are identified, contact precautions need to be put in place. As the prognosis is clearly dependent on a timely diagnosis and start of therapy, a quick lean diagnostic workup with an early lumbar puncture and targeted empiric therapy are the goal.

## Flow chart SOP emergency workup in patients with suspected acute bacterial meningitis (Fig. 1)

### Clinical presentation of patients with suspected acute bacterial meningitis

In patients with a combination of fever, headache, neck stiffness or altered level of consciousness, acute bacterial meningitis needs to be one of the top considered differential diagnosis. Of importance, single typical symptoms are absent in half of the patients. Thus, the clinical diagnosis cannot be ruled out only because the classical triad of fever, neck stiffness and impaired consciousness is not present. A hemorrhagic rash is more common in meningococcal meningitis, but it is not specific for an infection with *N. meningitidis*. Other less common clinical signs in bacterial meningitis are vomiting, photophobia, seizures, cranial nerve palsies, and hearing loss. Kernig and Brudzinski’s signs are not helpful to diagnose bacterial meningitis as their sensitivities and specificities are low.

**Fig. 1:**
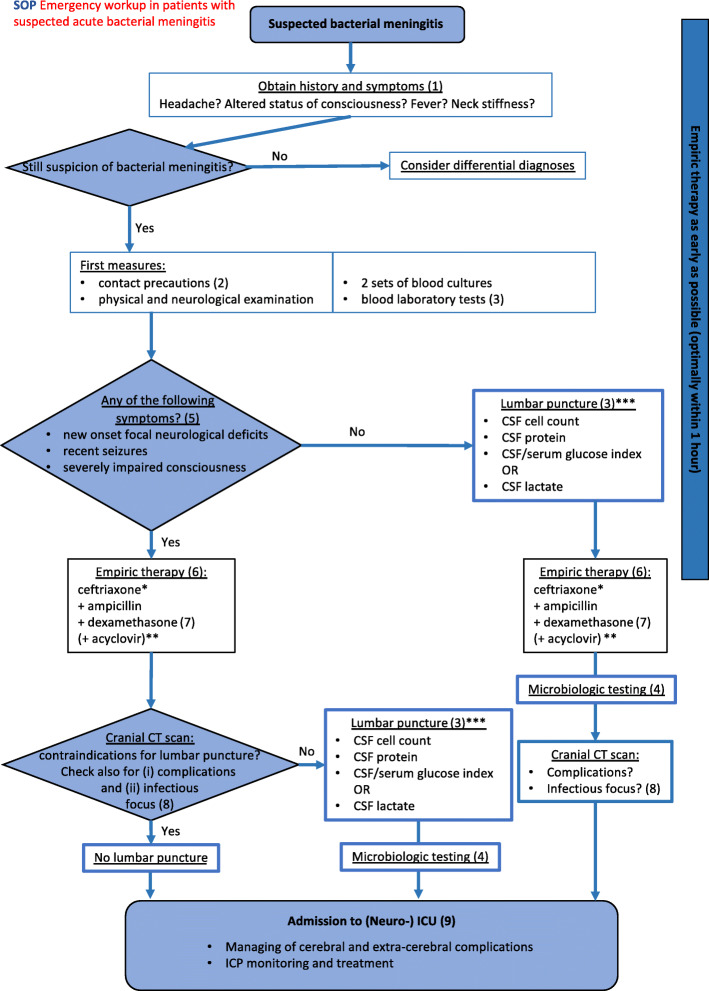
Clinical pathway for a patient with suspected bacterial meningitis in the emergency department. * in countries with a critical occurrence of cephalosporine-resistant *S. pneumoniae,* vancomycin or rifampicin are added to the antibiotic regimen (see recommendations of local authorities). ** if herpes simplex virus encephalitis is among the possible differential diagnoses *** general contraindications for lumbar puncture such as bleeding disorders need to be considered before lumbar puncture

According to studies on more than 1000 patients with bacterial meningitis, the most common symptoms in adult patients presenting with bacterial meningitis are fever (77%), headache (83–87%), neck stiffness (75–83%) and altered level of consciousness (69%) [5–7]. In recent studies from the Netherlands, France, Spain, Iceland and Denmark, only 41–51% of patients with bacterial meningitis showed the classical triad of fever, neck stiffness and altered mental status when presenting to the emergency department [5, 7, 8]. Especially in immunocompromised patients, single or several symptoms can be absent, as in immunocompromised patients with bacterial meningitis, only 36% showed the classical triad and 8% even displayed neither symptom of the triad [6]. The assessment of Kernig and Brudzinski sign is not of significant value in the detection of patients with bacterial meningitis.

- Classical symptoms of bacterial meningitis: fever, headache, neck stiffness, and altered level of consciousness
- Rash in 25% of patients with acute bacterial meningitis (68% in meningococcal meningitis)
- CAVE: Single or several classical symptoms can be absent, especially in immunocompromised patients
- Other common clinical symptoms: vomiting, photophobia, seizures, cranial nerve palsies, hearing loss

### Contact precautions

Whenever *N. meningitidis* is considered among the possible causative pathogens in patients with suspected bacterial meningitis, the patient needs to be isolated in a single room. Face masks, gloves, and gowns should be worn at room entry. If the patient leaves the room (e.g. for imaging or transfer to a patient ward), he/she should wear a facial mask. To avoid a delay in contact precautions and to protect other patients in the waiting room, these contact precautions should all be applied immediately to any patient presenting to the emergency room with the differential diagnosis of acute bacterial meningitis, ideally directly upon triage. In regard to an infection risk with meningococci, the droplet precautions should only be terminated within the first 24 h if bacterial meningitis is no longer suspected (e.g. after thorough history taking and clinical assessment by a physician), if the diagnosis is ruled out or in case of a persistent suspicion for bacterial meningitis with negative microbiologic workup for *N. meningitidis* AND another causative pathogen than *N. meningitidis* was found or is likely (e.g. in patients with a very clear parameningeal focus). If all microbiologic tests were found negative in a patient with CSF findings consistent with bacterial meningitis, contact precautions should be kept in place until 24 h after the initiation of an effective antibiotic therapy. Whenever *N. meningitidis* was identified in the index patient, adults with contact should receive chemoprophylaxis with ciprofloxacin (500 mg p.o., once), ceftriaxone (250 mg i.m., once) or rifampicin (600 mg i.v., 2x/day for 2 days). Chemoprophylaxis should be applied to contact persons within 24 h of exposure. Close household contacts should be vaccinated according to the serotype that was identified in the index patient.

- Establish contact precautions in suspected bacterial meningitis upon triage.
- After first medical assessment of the patient, an individual decision on further contact precautions needs to be made. If an infection with *N. meningitidis* is among the differential diagnosis, contact precautions need to be kept in place.
- Contact precautions need to be upheld until a minimum of 24 h after the first dosage of antibiotics.
- If *N. meningitidis* is identified, chemoprophylaxis should be offered to all persons with possible droplet contact.

### Laboratory workup

All patients with suspected bacterial meningitis should receive a basic laboratory panel including blood leukocyte counts, serum C-reactive protein CRP, and serum procalcitonin PCT. However, leukocyte count and CRP are quite unspecific. One important early biomarker for bacterial infection is serum procalcitonin (PCT): A meta-analysis with over 2000 patients found 95% sensitivity and 97% specificity for serum procalcitonin to distinguish bacterial from viral meningitis [9].

The most important workup on the way to establish (or rule out) the diagnosis of acute bacterial meningitis is the investigation of the CSF. Thus, a lumbar puncture needs to be performed and leukocyte counts in the CSF (including cell differentiation), CSF protein, CSF/serum glucose ratio or CSF lactate should be investigated. CSF pleocytosis with a predominance of polymorphonuclear leukocytes, elevated CSF protein and an increased lactate strongly indicates acute bacterial meningitis (Table 1). Single parameters can be in normal limits. If all of the above-mentioned CSF parameters are within normal limits, acute bacterial meningitis is ruled out.

**Table 1:** Typical CSF parameters in meningitis (according to [3])

	Bacterial	Viral	Tuberculous
**Cell count**	> 1000/μl^a^	< 1000/μl^b^	< 1000/μl
**Cytology**	Polymorphonuclear	Lymphocytic^c^	Mixed
**CSF/serum glucose index**	Decreased	Normal	Decreased
**Lactate**	> 3,5 mmol/l	< 3,5 mmol/l	> 3,5 mmol/l
**Protein**^**d**^	> 100 mg/dl	< 100 mg/dl	> 100 mg/dl

^a^Less than 1000 cells/μl in 1/3 of patients with bacterial meningitis

^b^In patients with Herpes virus encephalitis, cell counts in the initially assessed CSF can be within normal limits

^c^Polymorphonuclear cells usually dominate in patients with enteroviral meningitis and in the initial stage of viral meningitis

^d^Indicating blood-CSF barrier disruption

Classic findings in the CSF indicating bacterial meningitis are a pleocytosis of mostly polymorphonuclear leukocytes > 1000/μl, a low CSF-serum glucose index < 0,3 or an increase in CSF lactate, and an elevated protein concentration (> 100 mg/dl) [10]. However, in a cohort study from the Netherlands from 2006 to 2014 including over 1400 patients with bacterial meningitis, only 66% showed pleocytosis of over 1000 cells/μl while 11% had less than 100 cells/μl. Yet, in 96% of all patients with bacterial meningitis, at least one of the above described CSF findings (namely pleocytosis, elevated CSF protein or glucose CSF-serum index < 0,3) was present [11].

- Serum CRP and leukocytes are usually elevated in acute bacterial meningitis.
- Elevated serum procalcitonin is a helpful marker to distinguish bacterial from viral meningitis.
- Use of CSF procalcitonin is not recommended.
- CSF pleocytosis with > 1000 cells /μl, elevated CSF protein, and increased CSF lactate strongly indicate acute bacterial meningitis.
- CAVE: CSF pleocytosis < 1000/μl is found in one third of patients with bacterial meningitis in the CSF obtained by the initial lumbar puncture.

### Ancillary microbiologic tests

If CSF white cell count, CSF/serum albumin ratio and CSF/serum glucose ratio or CSF lactate are consistent with the diagnosis of bacterial meningitis, the following microbiologic tests with the goal to find the bacterial pathogen are recommended: First, two sets of blood cultures (each aerob and anaerob) should be investigated. Next, a gram stain should be done from CSF and CSF cultures should be initiated. If a pathogen cannot be detected by gram stain, rapid multiplex PCR testing seems of benefit. The use of latex agglutination tests can be considered in the case of a negative gram stain and a negative multiplex PCR or if multiplex PCR is not available. It is important to understand that any negative test does not rule out an infection by the pathogen that was covered by the test used (e.g. contact precautions cannot be stopped only because of a negative gram stain or a negative *N. meningitidis* PCR result). If the combination of CSF parameters (cell count, protein, CSF/serum glucose or serum lactate) shows a diagnosis of meningitis but is not typical for a bacterial infection, further tests might be indicated depending on the clinical syndrome and local predominant infections (e.g. PCR testing for Herpes simplex Virus encephalitis or serology for tick borne encephalitis in endemic regions).

CSF culture is perceived as “gold standard” of diagnostic tests detecting bacterial meningitis. However, culture results take time and can, as discussed above, be negative, e.g. when patients received antibiotic therapy before lumbar puncture. CSF gram stain is a quick and easily performed test of low cost that has a high yield in pneumococcal meningitis (90%) and meningococcal meningitis (70–90%) but is less often positive in *L. monocytogenes* meningitis (25–35%) and *H. influenzae* meningitis (50%) [1]. With CSF latex agglutination test, bacterial antigens can be detected rapidly, yet its sensitivity varies between 22 and 100% depending on tested microorganism and further decreases when the patient was pretreated with antibiotics [4, 12]. Another alternative method of pathogen detection is CSF PCR. Sensitivities of CSF PCR range from 79 to 100% for *S. pneumoniae*, 91–100% for *N. meningitidis*, 67–100% *for H. influenzae* and specificities of 95–100% [13]. Recently developed test kits allow PCR testing with 2 h and, furthermore, are set up for simultaneous testing of multiple common viral and bacterial pathogens that can cause meningitis. A meta-analysis of 8 studies and 3059 patients on a possible benefit of using multiplex PCR testing in suspected meningitis suggested a high sensitivity (90%) and specificity (97%) [14].

- Order microbiologic tests if the suspicion of bacterial meningitis persists after evaluation of CSF white cell count, CSF protein and CSF/serum glucose ratio or CSF lactate.
- Two sets of blood culture (one set: aerob and anaerob) should be evaluated.
- Obligatory CSF tests in bacterial meningitis: CSF gram stain and CSF culture.
- In case of a negative CSF gram stain: multiplex PCR from CSF (if available).
- Consider further tests (latex agglutination, PCR) in individual patients
- CAVE: specific pathogens cannot be ruled out as causative solely by negative microbiologic test results.

### Time of lumbar puncture and cerebral imaging

Patients with suspected acute bacterial meningitis should receive imaging of the brain (by computed tomography) prior to lumbar puncture in case of a new focal neurologic deficit, recent epileptic seizures (within days before presentation) or severely impaired consciousness. In all other cases, lumbar puncture is recommended without prior cerebral imaging. In case of a delay of lumbar puncture due to prior cerebral imaging or any other reason, antibiotic therapy should be initiated immediately, before imaging and lumbar puncture. In any case, taking blood cultures before empiric therapy is important.

If space-occupying lesions such as brain abscess or subdural empyema are present in a patient with suspected bacterial meningitis, risk of brain herniation due to LP is increased [13]. A study on 235 patients with suspected acute bacterial meningitis, space-occupying cerebral lesions were found in patients with impaired consciousness, epileptic seizures, and focal neurologic deficits [15]. Overall, it appears save to seek for lumbar puncture in patients without severely impaired consciousness, recent epileptic seizures or new focal neurologic deficits. Several studies demonstrated that performing a computed tomography before lumbar puncture is associated with a delay in the lumbar puncture, a delay in the start of antibiotic therapy, and poor outcome. Thus, if a computed tomography is performed before lumbar puncture, leading to a significant delay, antibiotics should be given before the computed tomography scan.

- Draw blood cultures (2 pairs)
- Seek for early lumbar puncture in patients with suspected bacterial meningitis
- Cerebral imaging before lumbar puncture is needed in patients with (i) new onset focal neurologic deficits, (ii) recent epileptic seizures, and/or (iii) severely impaired consciousness.
- Lumbar puncture without prior cerebral imaging if clinical signs for increased intracranial pressure are absent.
- In case of a delay of lumbar puncture start antibiotic therapy before cerebral imaging.

### Empiric antibiotic therapy

For empiric therapy, the German guidelines recommend empiric therapy with ceftriaxone plus ampicillin (Table 2). For areas with a higher resistance rate of *S. pneumoniae* to 3rd generation cephalosporines (e.g. southern Europe, France, Spain, Northern America), an additional therapy with vancomycin or rifampicin is recommended [4]. If there is a possibility of herpes simplex virus encephalitis, the addition of acyclovir (3x10mg/kg body weight / day) is recommended. Therapy should be adapted after a causative pathogen was identified. Several studies have revealed that a delay in antibiotic treatment in patients with acute bacterial meningitis leads to deterioration of prognosis. According to ESCMID guideline, empiric treatment is recommended to be started on clinical suspicion within 1 h after arrival at the hospital [4].

**Table 2:** Empiric antibiotic therapy in acute bacterial meningitis (according to [3, 4])

Clinical Situation	Empiric antibiotic therapy	Dosage
Community-acquired	ceftriaxone plus ampicillin^a^ (plus vancomycin^b^)	2x2g/day i.v. 6x2g/day i.v. 2x1g/day i.v. (check serum concentration)
Nosocomial	Meropenem plus vancomycin alternative option: ceftazidime plus vancomycin	3x2g/day i.v. 2x1g/day i.v. (check serum concentration) 3x2g/day i.v. 2x1g/day i.v. (check serum concentration)
Immunocompromised patients	ceftriaxone plus ampicillin plus vancomycin^b^	2x2g/day i.v. 6x2g/day i.v. 2x1g/day i.v. (check serum concentration)

^a^In case of penicillin allergy, the use of meropenem is recommended instead (3x2g/day i.v)

^b^In case of a high prevalence of *S. pneumoniae* that are resistant to 3rd generation cephalosporines (see recommendations of local authorities). As an alternative to vancomycin, rifampicin (1x600mg/day i.v.) can be administered

The most common pathogen causing bacterial meningitis in adults is *S. pneumoniae.* In a Dutch cohort study from 2006 to 2014 including more than 1400 cases of bacterial meningitis, 72% were due to *S. pneumoniae* [11]. Other common causative pathogens were *N. meningitidi*s (11%), *L. monocytogenes* (5%) and *H. influenzae* (3%) [11]. Although *L. monocytogenes* usually occurs only in elderly patients or patients with immunosuppression, the immune status of the patient is frequently unclear at the time when the patient first presents to the emergency room. Therefore, e.g., the guideline of the German Neurological Society (DGN) recommends ampicillin for initial empiric therapy in all adult patients with suspected bacterial meningitis [3].

In practice, differentiation between bacterial meningitis and viral meningitis can be difficult, especially before a pathogen is identified. If that is the case, the most common treatable causative pathogen of viral encephalitis, namely Herpes simplex Virus Type 1 needs to be treated empirically as well.

Dosages of therapeutic drugs should be adapted to weight and kidney function of each individual patient. The duration of antibiotic treatment is recommended for (7-)14 days depending on causing microorganism, resistance and clinical condition of the patient. In cases of infection with *L. monocytogenes*, antibiotic therapy is recommended for at least 21 days [4].

- Empiric antibiotic therapy in community-acquired bacterial meningitis: ceftriaxone plus ampicillin.
- Add vancomycin or rifampicin in areas with significant rates of pneumococcal resistance against 3rd generation cephalosporines (please check recommendations from local scientific authorities).
- Add acyclovir if herpes virus encephalitis is a differential diagnosis.

### Adjunctive treatment with dexamethasone

In countries with a high standard of medical care, empiric therapy in patients with suspected bacterial meningitis should include adjunctive therapy with dexamethasone (4x10mg / day i.v.). Dexamethasone should optimally be given with the first dosage of antibiotics. If antibiotic therapy has already been initiated, the start of dexamethasone therapy within 4 h seems to be justified [4]. If *S. pneumoniae* or *H. influenzae* is identified as the causative pathogen, therapy with dexamethasone should be upheld for a total of 4 days. If other pathogens are identified, therapy with dexamethasone should be discontinued. If dexamethasone is used, therapy with vancomycin should be replaced, e.g. by rifampicin, as dexamethasone has been reported to impair vancomycin’s ability to transfer into the CSF.

- In countries with a high level of medical care: empiric adjunctive therapy with dexamethasone is recommended.
- Continue dexamethasone if *S. pneumoniae* or *H. influenzae* is identified

### Further diagnostic investigations

Important intracranial complications that need to be detected early, should not be missed in the emergency department in patients with bacterial meningitis. Thus, on admission day, patients should receive cerebral imaging (computed tomography in most cases) to look for cerebral oedema, focal brain swelling, and hydrocephalus. If such complications are detected, proper treatment in accordance with the clinical findings in the patient is indicated (e.g. placement of an external ventricular drain in case of clinically relevant hydrocephalus). If seizures are reported, antiepileptic therapy should be started.

A search for an infectious focus is crucial in patients with bacterial meningitis. In addition to a thorough neurological and medical physical examination, patients should be examined for an infection of the ear (otitis media/mastoiditis) and the sinuses (sinusitis), possibly supported by bone windows on computed tomography that focus on the respective areas. On computed tomography cerebral imaging, one focus should be kept on the possibility of intracranial air as a sign of cerebrospinal fluid leakage. In case of an ENT focus, surgical treatment should be carried out immediately (optimally within the first 24 h). If clinical signs for another infectious focus are present, special workup is required (e.g. transoesophageal echocardiography for suspected endocarditis or spinal magnetic resonance imaging for spondylodiscitis or spinal abscess).

- Search for intracranial complications on computed tomography – look for signs of hydrocephalus, brain oedema, and/or focal brain swelling.
- In case of a clinically relevant hydrocephalus, place an external ventricular drain (EVD).
- Search for infectious focus: common ENT foci are mastoiditis and sinusitis. Consider endocarditis in case of a heart murmur, Osler spots, and septic embolic cerebral ischemia. In case of reported back pain, consider spinal imaging.
- Seek for early surgical therapy if an ENT focus is found.

### Further patient management

Patients with acute bacterial meningitis should initially be treated at an intensive care unit with expertise in the treatment of severe neurological diseases. Patients with bacterial meningitis have an increased risk of complications such as hydrocephalus, subdural empyema or intracerebral abscess [16]. Patient management may include mechanical ventilation, treatment of elevated ICP, circulatory support or medical treatment of coagulopathy or hyponatremia warranting treatment in intensive care units (ICU) [12]. As complications might develop during the first days of therapy, initial treatment at an intensive care unit is recommended. A study from Sweden demonstrated, that among patients with severe bacterial meningitis (low Glasgow Coma Score at the time point of admission) that were treated at an ICU, patient outcome was best at an ICU with expertise in the treatment of severe neurological diseases and the ability to invasively monitor and treat increased intracranial pressure [17].

- Initial treatment at an intensive care unit with neurological expertise is recommended.

## Conclusions

Suspected acute bacterial meningitis is an emergency and requires attention with high priority in the emergency department to ensure a quick workup and early start of therapy. A targeted diagnostic workup with a focus on a timely lumbar puncture is crucial. If lumbar puncture is delayed for any reason, empiric therapy with ceftriaxone, ampicillin, and dexamethasone (plus rifampicin or vancomycin) should be started immediately, before cerebrospinal fluid is obtained. On the day of admission, a special focus also needs to be put on the detection of severe intracranial (and systemic) complications and on the search for the infectious focus. Admission of patients with bacterial meningitis to an intensive care unit is highly recommended.

## Abbreviations

CNS: Central nervous system
CRP: C-reactive protein
CSF: Cerebrospinal fluid
H: Haemophilus
ICP: Intracranial pressure
ICU: Intensive care unit
L: Listeria
MRI: Magnet resonance imaging
N: Neisseria
PCR: Polymerase chain reaction
PCT: Procalcitonin
S: Streptococcus
